# Piezoelectric Multi‐Channel Bilayer Transducer for Sensing and Filtering Ossicular Vibration

**DOI:** 10.1002/advs.202308277

**Published:** 2024-02-21

**Authors:** Muhammed Berat Yüksel, Ali Can Atik, Haluk Külah

**Affiliations:** ^1^ Department of Electrical and Electronics Engineering Middle East Technical University (METU) Universiteler Mah. Dumlipinar Blv. No:1 Ankara 06800 Turkey; ^2^ METU MEMS Center Mustafa Kemal Mah Dumlupınar Bulvarı No: 280 Ankara 06350 Turkey

**Keywords:** fully implantable cochlear implant, MEMS, piezoelectric transducer

## Abstract

This paper presents an acoustic transducer for fully implantable cochlear implants (FICIs), which can be implanted on the hearing chain to detect and filter the ambient sound in eight frequency bands between 250 and 6000 Hz. The transducer dimensions are conventional surgery compatible. The structure is formed with 3  × 3 × 0.36 mm active space for each layer and 5.2 mg total active mass excluding packaging. Characterization of the transducer is carried on an artificial membrane whose vibration characteristic is similar to the umbo vibration. On the artificial membrane, piezoelectric transducer generates up to 320.3 mV_pp_ under 100 dB sound pressure level (SPL) excitation and covers the audible acoustic frequency. The measured signal‐to‐noise‐ratio (SNR) of the channels is up to 84.2 dB. Sound quality of the transducer for fully implantable cochlear implant application is graded with an objective qualification method (PESQ) for the first time in the literature to the best of the knowledge, and scored 3.42/4.5.

## Graphical abstract

This study presents a middle ear implantable transducer for fully implantable cochlear implants. The transducer`s dimensions are compatible with routine cochlear implant procedures. The multi‐channel system covers the daily acoustic band with a high signal‐to‐noise ratio. Objective audiological evaluations of speech signals underscore the encouraging results, affirming the feasibility of the middle ear implantable multi‐channel transducer for the first time.

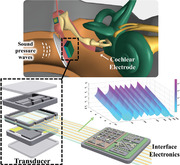

## 1 Introduction

The world “celebrates” the one‐millionth cochlear implant user,^[^ ^1^ ^]^ while the World Health Organization estimates one in every ten people will have different levels of hearing loss by 2050.^[^ ^2^ ^]^ This analogy reveals the cruciality of the need for technological improvement in hearing prostheses. Cochlear implants (CIs) are medical devices that can restore the hearing functionality of individuals with severe‐to‐profound sensorineural hearing loss. Conventional CIs consist of implanted (cochlear electrode, receiver coil) and external (the battery, microphone, sound processor, and transmitting coil) that expose CI to external trauma, effects of head movement, and gravity. Therefore, the outer part prevents the implant system from being used during many physical activities, such as participating in water sports, showering, or sleeping. In addition, damaged wires, cables, speech processors, and transmit coils are a source of problems and expenses for the patient and manufacturer by causing excessive downtime and auditory deprivation.^[^ ^1^, ^3^, ^4^ ^]^

CIs in the research phase can perform relatively well in challenging speech tests and enable high‐quality music perception, while commercial CI's performance is 70–80% correct sentence recognition in a quiet environment.^[^ ^1^ ^]^ In conventional CI, the natural hearing mechanism is bypassed from the pinna to the cochlea; and natural signal processing on the pinna, ear way, eardrum, and ossicle movements is neglected. This neglected chain amplifies specific frequencies and controls the pressure on the oval window. Bypassing these peak points of the complex biological systems degrades the performance of the restored hearing. Innovations in sensor systems could open up an opportunity to use natural hearing mechanisms.

Cochlear implant technology is the leader in implantable electronics. However, technological development of FICIs has lagged, and other fields have started to catch up in recent years. One of the main obstacles to FICIs is an implantable sound detector. Advancements in microelectromechanical systems (MEMS) technology underpin the fabrication of small, high‐performance transducers to convert ambient vibration to electrical potentials and enable the emerging concept of FICIs. Detecting the ambient sound via vibration of the hearing chain with ultra‐low‐power interface electronics and implantable battery allows discarding of outer components. In recent years, this idea has been proposed by different companies and research groups. While some companies have ongoing clinical trials^[^ ^5^ ^]^ and Food and Drug Administration A Investigational Device Exemption approvals^[^ ^6^ ^]^ for the whole system, in the literature, studies focus on individual components of the FICI systems, such as implantable acoustic sensors, low‐power interface electronics, cochlear electrode designs, and packaging of the systems. Briggs et al. provided a basis for totally implantable systems by performing a clinical trial on three adults.^[^ ^7^ ^]^ They designed a totally implantable system with an electret microphone, application‐specific integrated circuits, and a special package for subcutaneous operation.

The major bottleneck in developing the FICI system is detecting ambient sound. State‐of‐the‐art technology in the remaining parts of the system has reached satisfactory performance. There are ultra‐low power stimulation interface circuits, multi‐mode wireless power transfer circuits, and highly efficient power management circuits. Performance loss in these subsystems will only affect the system's battery life. On the contrary, the performance of sound detection will define the quality of sound perception.

In order to construct implantable acoustic transducers, capacitive, and piezoelectric techniques have been researched in the literature. Young et al. placed a MEMS capacitive accelerometer‐based transducer to the umbo. However, sensor output is considerably low, and their readout circuit consumes too much power (≈4.5 mW), which limits the implantable system's battery usage of the implantable system.^[^ ^8^ ^]^ In sound detection, the piezoelectric effect is the most appropriate solution for low‐power systems due to its high‐voltage outputs and not requiring a separate electrical source to initiate the signal transduction process. Further, piezoelectric methods enable improving the output level by increasing the stress level on the structure, thus increases the viability of the piezoelectric approach for implanted applications. These key parameters enable the biomedical implementation of MEMS piezoelectric acoustic devices in different areas. The middle ear and intracochlear^[^ ^9^ ^]^ implantations using piezoelectric technology have been investigated in this manner. Single channel piezoelectric vibration sensor for cochlear implants has been designed by Hake et al., and they concluded that single channel system could not achieve minimum detectable signal levels.^[^ ^10^ ^]^ Even if the signal level is improved, a single channel system will require an additional filtering system, which increases the power consumption of the interface electronics. Udvardi et al. presented a 16‐channel spiral‐shaped AlN piezoelectric cantilever array system for middle ear implantation. They only cover the hearing band's lower part (300–700 Hz) and generate only 9.6 mV under 1 g excitation.^[^ ^11^ ^]^ Gao et al. proposed a floating piezoelectric microphone,^[^ ^12^ ^]^ and Jia et al. tested a biocompatible encapsulated version of the floating piezoelectric microphone on a fresh human cadaver.^[^ ^13^ ^]^ Jang et al. designed a piezoelectric‐based artificial basilar membrane that includes ten channels to filter ambient sound into frequency bands.^[^ ^14^ ^]^ Then, Jang et al. designed the artificial basilar membrane based on an AlN cantilever array with an 8‐channel distributed on the band between 2.92 and 12.6 kHz and generated 4.06 mV at 7.04 kHz under 101.7 dB SPL.^[^ ^15^ ^]^ The frequency range of these structures did not cover daily sounds; therefore, it is unsuitable for hearing applications. Zhao et al. developed an AlN intracochlear transducer consisting of 4 cantilever beams array.^[^ ^16^ ^]^ This offbeat method can generate up to 79.7 µV under 95 dB SPL in a guinea pig, where it requires dedicated sensing and amplification stages that can be problematic for low‐power applications. A very sensitive piezoelectric mobile acoustic sensor for machine learning biometrics was reported by Wang et al. This flexible acoustic sensor has seven channels at biomimetic frequency ranges and was developed for mobile applications. Compared to earlier studies, this sensor's sensitivity (52 mV Pa^−1^) and signal‐to‐noise ratio (up to 92 dBV) are superior.^[^ ^17^ ^]^ The structure, however, covers a significantly larger area than the middle ear's accessible space.

**Figure 1:**
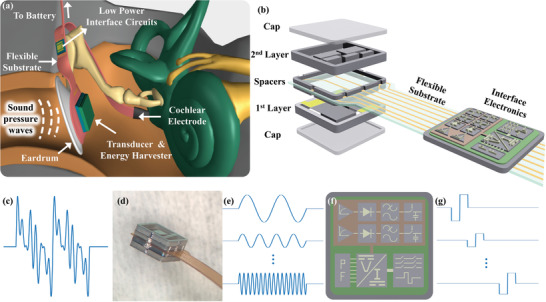
Fully implantable cochlear implant concept illustration. a) Illustration of the system on ear anatomy. b) Bilayer transducer and interface electronics. c) Ambient sound. d) Fabricated transducer. e Filtered signal. f) Interface electronics and subblocks. g) Generated stimulation pulses.

Our group has proposed a challenging approach to the self‐powered fully implantable cochlear implant concept. This concept, FLAMENCO: A Fully‐Implantable MEMS‐Based Autonomous Cochlear Implant, includes a multi‐channel acoustic transducer,^[^ ^18^, ^19^, ^20^ ^]^ a piezoelectric energy harvester,^[^ ^21^, ^22^ ^]^ an interface circuit,^[^ ^23^ ^]^ and a commercial cochlear electrode.^[^ ^24^, ^25^ ^]^ To comply with the FICI standards, the entire system can be implanted inside the middle ear cavity, collect the ambient sound by using the natural hearing mechanism, and filter the signal into frequency bands to stimulate the auditory nerves (**Figure** **1**). In this system, electrical stimulation of the fully implantable CI is defined by the cantilevers via the piezoelectric effect. Piezoelectric cantilevers should be able to activate low‐power interface electronics and cover the acoustic band with enough channels in a space as small as the middle ear.

This study presents a practical approach for implanting the MEMS sensor into the middle ear, during a routine cochlear electrode array surgery via posterior tympanotomy. This conventional technique enables access to the middle ear and provides strategic anchoring points on the hearing chain for sensor clamping. This approach not only streamlines the surgical process but also offers the possibility to enhance cochlear implantation functionality by incorporating a MEMS sensor. Thus, in this study, the multi‐channel bilayer structure and its fabrication are optimized for FICI applications. Parameters of each channel were specified for each frequency range by considering their structural strength, voltage output, and filter characteristics. The functionality of the transducer array was validated by subjecting it to single‐tone, speech, and emotional signals, on an artificial tympanic membrane with human‐like characteristics. Objective audiological evaluations underscore the encouraging results, affirming the feasibility of the middle ear implantable multi‐channel transducer for the first time.

## 2 Design and Experimental Section

### 2.1 Transducer Design

Designing a fully implantable system that was expected to continue operating inside the middle ear for life requires comprehensive analysis and reveals complex design parameters. Dimensions, frequency range, mass, number of channels, minimum detectable signal level, noise, power consumption, sensitivity, and output signal level were the main design parameters for an implantable transducer.

Sound detectors in the literature were aimed to be placed in the middle ear, intracochlear, and or subcutaneously implanted in the mastoid. Subcutaneous microphone performance was affected by the degradation of pressure level through the skin and internal body noise. The device's durability and the cochlea's dimensions were the main obstacles in intracochlear applications. Therefore, the appropriate location for the sound detector was the hearing chain to utilize vibration on the natural hearing mechanism. However, surgical techniques and accessible dimensions of the middle ear implicitly restrict the dimensions and output performance of the device. The middle ear has a limited volume, 1 cm^3^, and the average area on the eardrum was 9 × 10 mm^2^. All these possible areas and volumes could not be utilized in order not to dispatch the hearing chain to maintain natural hearing characteristics and to complete the surgery with minimum invasion. In the conventional CI surgery procedure, surgeons apply posterior tympanotomy,^[^ ^26^ ^]^ and the mean dimension of the opening, measured in the literature, was ≈4.7 mm.^[^ ^27^ ^]^ Inadequate large exposures of the ear may reduce the barrier to future infections. Therefore, the device must be minimized to avoid further complications. Furthermore, connection aperture, surgery tools, maneuver of the surgeon, range of motion, and possible differences between persons' anatomy were considered while defining the transducer`s maximum dimensions. In this study, 3.5 mm was defined as the maximum dimension for the structure, including frames, and packaging.

Individuals with normal hearing could perceive frequencies from 20 Hz to 20 kHz within a 0 to 140 dB SPL. These ranges were peak points of the evolution of the human race and cannot be replicated with today`s technology. However, the daily sound spectrum can still be recovered. The human voice ranges from 250 Hz to 4 kHz.^[^ ^28^ ^]^ The range between 4 and 6 kHz was also essential for hearing in a noisy environment, while environmental and internal body noises stay below the range.^[^ ^29^ ^]^

Natural hearing characteristic was affected by additional mass or force loading. In vivo studies show that adding mass greater than the umbo's total mass and the long malleus process, 25 mg, reduces the vibration amplitude by 3 dB.^[^ ^30^ ^]^ Therefore, it was crucial to keep the total mass low for sound detection quality and consider all additional packaged structures.

The organ of Corti consists of ≈3500 frequency selective inner hair cells to translate the vibrations into electrical signals. Conventional CIs mimic the operation by using up to 22 channels depending on the brand and sound processing strategy. The effect of the number of channels was investigated in the literature, and studies revealed that sound perception of the CI users significantly improves up to 8‐channel, then the correct perception rate saturates while increasing the channel numbers.^[^ ^31^, ^32^, ^33^ ^]^ Dimensions, mass, sensitivity, and other parameters were affected by the channel number. Therefore, defining the appropriate channel number was critical for the transducer.

Interface electronics process the output signal of the transducer to turn vibrations into biphasic stimulation waveforms. Interface electronics for fully implantable systems were aimed to consume ultra‐low power and extend the battery lifetime. For this purpose, they use low‐power CMOS processes that restrict the minimum input level of the circuit. Therefore, the output signal level of the transducer must exceed this minimum level to excite the circuit for neural stimulation. The interface electronic with the lowest power consumption requires a 100 µV minimum input level.^[^ ^23^ ^]^ In this amplitude‐modulated interface circuit (Figure 1f), the front‐end unit reduces power dissipation by integrating amplification and compression of the sensor output through an ultra‐low‐power logarithmic amplifier. Subsequently, the amplified signal underwent envelope detection and was directed to a voltage‐controlled current source, functioning as a reference for the generation of stimulation current. This generator incorporates a 7‐bit user‐programmable DAC, facilitating the configuration of stimulation levels for individual patient adaptation. The nerves receive stimulation through adjusted biphasic currents based on the continuous inter‐leaved sampling (CIS) stimulation strategy facilitated by a switch matrix. The entire stimulation system has been configured to accommodate a measured input dynamic range of up to 60 dB and an average stimulation current of up to 1 mA. The 8‐channel stimulation interface dissipates 472 µW power to achieve full functionality when stimulated by a simulated speech signal.^[^ ^23^ ^]^

A fully implantable cochlear implant system couldn't be completely achieved using any of the structures presented in the literature. However, they prove the availability of piezoelectric methods to sense vibration on the ossicular chain. In the development process of the bilayer multi‐channel sound detector, a thin film single‐channel sound detector system has been designed as a proof‐of‐concept for thin film applications.^[^ ^20^ ^]^ Then, an eight‐channel sound detector with thin film PLD‐PZT piezoelectric layers was designed. The dimensions of the sound detector were suitable for middle ear implantation. Correlations between the input and output waveforms of the speech test also demonstrated that this approach could overcome the bottlenecks of the middle ear implantable sound detectors.^[^ ^18^ ^]^ The sound detector presented in this study comprises the previous studies` outcomes and accommodates surgical operation methods. Available space in the middle ear could not be entirely utilized due to tissues, passing nerves, and long‐term patient health. Dimensions of the previous studies were not compatible with this approach and could bring considerable surgical intricacies, including the risk of trauma, and compromised structural integrity. Therefore, in this study, the dimensions to conventional surgery technique dimensions were restricted, which leads the investigation to a bilayer structure.A multi‐channel thin film PZT‐based piezoelectric transducer was designed and the outputs in the COMSOL model were calculated. This transducer satisfies FICI application specifications without the need for readout electronics.

Beam structures were capable of moving with high deflection, which caused strain on the fixed size. Piezoelectric films in the 31‐mode could utilize this strain to sense the vibration. However, resonance frequencies of simple beam structures within available dimensions were greater than the aimed frequency range and generated low output signals. Adding mass to the tip of the beam provides dense structures by enabling the reduction of the lengths of the beams and increases strain levels on piezoelectric film. Therefore, they could be applied to detect sound from middle ear vibrations while resonating in the hearing band. These resonance frequencies define the center frequencies of the frequency bands. The number of frequency bands and their center frequencies have been studied in the literature. Results prove that 8‐channel systems were the most convenient for low‐power applications, and frequencies were distributed linearly from 300 up to 1200 Hz and logarithmically above 1200 Hz in the daily sound signals range.^[^ ^34^ ^]^ Thin‐film PLD‐PZT (MESA+ Institute, Netherlands) has been chosen due to its higher piezoelectric properties to obtain a wide dynamic range.

The finite element analysis program COMSOL Multiphysics was used to model the structure to create the transducer under the specified constraints (number of channels, size, frequency range, mass, and output voltage). The meshing of the model affects the computational time and accuracy of the results. Accuracy was enhanced with smaller meshes. However, this method expands the computing time, particularly when the structure has a thin layer. Since metal and insulating layers' material characteristics and thicknesses barely affect the output characteristics, they were disregarded. Additionally, thin layers were unable to mesh with free meshes that have only two elements. Due to the lack of computation of the material's stress level, this pattern has lower accuracy. Therefore, the simulation model's mesh size was manually adjusted until it converged. During simulations, four main assumptions were applied: 1) Air damping was excluded, 2) Metal and isolation layer was omitted, 3) ideal load was connected, and 4) electrical damping due to the input characteristics of the interface circuit wasn`t calculated. Vibration characteristics were observed under constant acceleration during simulations to achieve the necessary output signal for neural stimulation via low‐power interface electronics. Results of in vitro tests on displacement properties of the umbo were utilized as a guide for acoustic performance calculations.^[^ ^35^ ^]^

The limited volume and area of the middle ear were resolved with a bilayer beam array design. Bilayer concepts arise due to two main facts. The limited dimension of the middle ear opening during the surgery restricts the design, and low‐frequency channels, especially the first channel, barely fit into the available length. The effort to reduce the lengths of the beams with specified frequencies for single‐layer application by reducing the thickness of the beam or using a narrower beam will let the system shrink but sacrifice the endurance of the structure. Therefore, channels couldn't be distributed on a single layer with the design restrictions. Stacking two layers forms a compact implantable system without sacrificing performance. To minimize the size of the sensor, channels were placed concerning their lengths. The first layer, 3‐channel, includes beams with 300, 1200, and 1600 Hz resonance frequency, while the second layer, 5‐channel, consists of 600, 900, 2200, 3200, and 4800 Hz channels, as shown in Figure 1. The arrangement of channels and the layered structure were not the sole aspects considered in this concept. During the system design, all channel parameters were configured individually for each resonance frequency. In contrast to this prior research, where all channels shared the same width, this variation in channel dimensions led to substantial differences in structural strength, noise levels, output performance, and filter characteristics when compared to the findings presented in this study. Moreover, beam thickness was selected as 10 µm to arrange the output and stress levels on beams. The final selection of the design parameters is represented in **Table** **1**. This new generation design has a mass of 5.3 mg that stays well below the loading limit of the excess mass on the ossicles for the vibration and an active volume of 3 × 3 × 0.36 mm for each layer, excluding packaging and frames. The simply packaged structure has a 3.5 × 3.5 × 1.52 mm total size and 20.1 mg total mass. The simulation model was explained, and the simulation flow was shared in the Supplementary Information. Details and consistency of the simulation results have also been studied in ref. [18].

**Table 1:** Design parameters and output performance of the 8‐channel transducer.

Channel number	Length [µm]	Width [µm]	Mass Length [µm]	Piezo Length [µm]	Freq. [Hz]	Output Level [mV_pp_]	Sens. [mV Pa^−1^]	SNR [dB]
1	2900	1300	1400	700	326	141.6	70.8	66.8
2	2100	1300	1100	500	616	296.8	148.4	74.7
3	1800	1250	600	440	870	163.4	81.7	75.4
4	1500	1250	500	425	1148	115.7	57.8	79.4
5	1300	1250	450	325	1479	138.6	69.3	81.0
6	1100	900	400	275	2030	160.7	80.3	74.2
7	900	900	300	225	2929	320.3	160.2	84.2
8	800	800	250	175	4615	36.1	18.0	81.8

### 2.2 Fabrication

**Figure 2:**
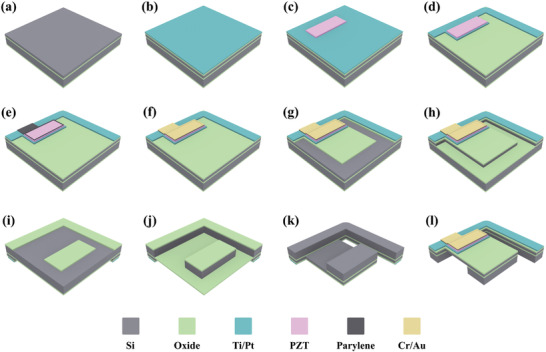
Fabrication process flow of the bilayer transducer. a) SOI wafer, b) Thermal oxidation and bottom electrode deposition, c) PLD‐PZT deposition and patterning, d) Bottom electrode patterning, e) Parylene‐C deposition and patterning, f) Top electrode deposition and patterning, g) Thermal oxide patterning by RIE, h) Beam formation by DRIE, i) Backside thermal oxide patterning by RIE, j) Backside structure formation by DRIE, k) Buried oxide removal with RIE, and l) fabricated beam structure.

**Figure** **2** shows the fabrication process. Silicon on Insulator wafers (p‐type, 10 µm Si device layer, 1 µm buried oxide, and 350 µm handle layer, 100 mm diameter) were used as a substrate to fabricate bilayer transducers. A 500 nm‐thick SiO_2_ layer was produced by thermal oxidation and used for lateral insulation between the electrodes via device layer. A Ti/Pt layer (20/100 nm) was sputtered to form a bottom electrode. A 14 nm lanthanum nickel oxide (LNO), seed/orientation layer, and 1µm‐thin PLD‐PZT were deposited by MESA+ Institute, Netherlands. PLD‐PZT, and LNO layers were patterned by wet etching. While patterning 100 nm Pt layer, fast and improved aqua regia etching developed by the group was used.^[^ ^36^ ^]^ Then, 20 nm Ti layer was patterned by wet etching. A 1 µm Parylene‐C layer was deposited by evaporation (SCS‐ PDS 2010 System) as a vertical isolation layer. Parylene‐C layer was patterned with RIE process using CF_4_ and O_2_ gases for top electrode openings on PLD‐PZT layer. A Cr/Au (30/400 nm) top electrode layer was deposited by sputtering and patterned using Transene Etchants. The remaining naked Parylene‐C was removed by RIE process while using the top electrode as a hard mask and completed the surface micromachining. Beams and mass structures were formed by device and handle layers of the SOI wafer, and buried oxide was used as a stop layer in DRIE processes. Thermal oxides and silicon layers were shaped by RIE and DRIE processes both on the front and back sides. Alumina wafer was temporarily bonded for the backside process by using Crystalbond 555 to the device side of the wafer after spray photoresist coating to protect the cantilever surface. After shaping the structure, the buried oxide layer was etched away by RIE, and temporary bonding was resolved in DMSO at 80 °C. Finally, the devices were released from the wafer by breaking thin bridges without dicing. Optical images of the fabricated layers are shown in Figures S6,S7 (Supporting Information). Spacers and caps for packaging the bilayer transducer were fabricated by using silicon wafers (p‐type, 200 µm, 100 mm diameter). A 500 nm oxide was deposited by PCVD and patterned by RIE. Then, structures were shaped by DRIE. Finally, a 500 nm oxide was deposited again for isolation.

### 2.3 Transducer Characterization

**Figure 3:**
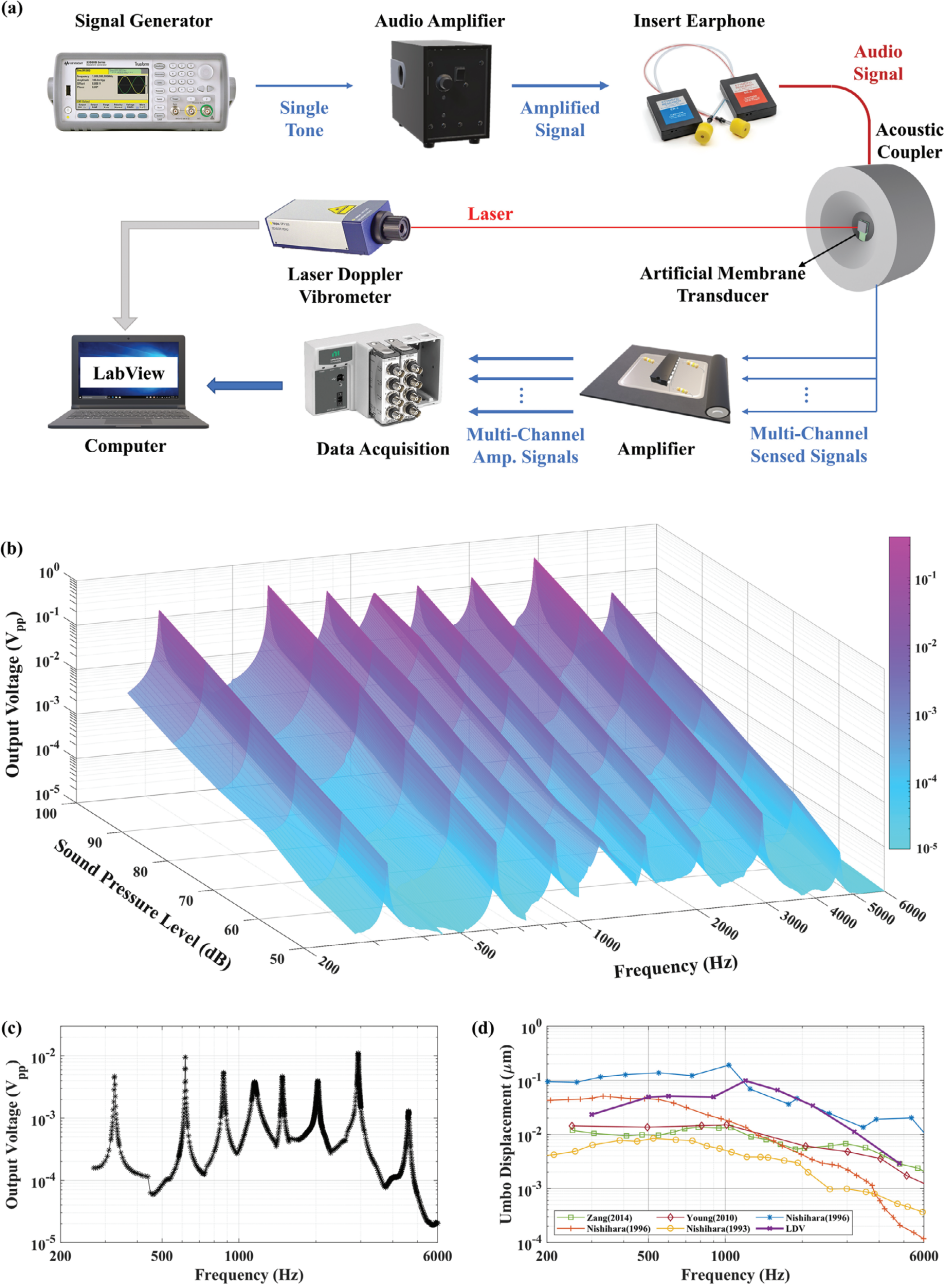
Acoustic characterization of transducer. a Schematic of the test setup. b Output waveforms of 8‐channel system under 50 to 100 dB SPL excitation in the hearing band. c 8‐channel output characteristic of the transducer under 70 dB SPL excitation. d Vibration characteristic of the artificial membrane and its comparison with the measured vibration amplitudes of the umbo under 80 dB excitation in the literature.

The electrical characteristics of the multi‐channel thin‐film piezoelectric transducer were measured by a Precision LCR Meter (Keysight E4980A). The resonance characteristics of the transducer were substantially consistent with the design. Microfabrication defects and residual stresses in the structure cause slight shifts in the resonance characteristic. Then, the sound detector was characterized acoustically with a custom‐made auditory canal and eardrum as a test environment, as shown in **Figure** **3**a. The physical model of the human tympanic membrane was reproduced in previous studies of the group.^[^ ^37^ ^]^ PDMS‐based artificial membrane has a resonance characteristic ≈1 kHz, similar to the tympanic membrane characteristic, and vibration amplitudes were close to previous reports. The sound detector was placed on the artificial membrane, which provides a medium for conversion from acoustic pressure to mechanical vibration, and electrical connections were carried on a flexible substrate. The artificial membrane was fixed in an acoustic coupler and excited with an insert earphone (Etymotic Research, ER‐2). An audio amplifier (DENON PMA520) was used to boost the pure tone sine signal produced by the signal generator (Keysight 33522B). A sound level meter (IEC 651 Type II) and a 2‐cc acoustic coupler were used to calibrate and arrange the excitation level. The generated electrical signal was amplified by using an instrumentation amplifier (INA121P), powered by a battery to minimize electrical noise. Data Acquisition Board (DAQ – NI cDAQ‐9174) and LabView software detected and recorded the amplified signal. The vibration level of the membrane was monitored by using Laser Doppler Vibrometer (LDV, Polytec, and NLV‐2500).

**Figure 4:**
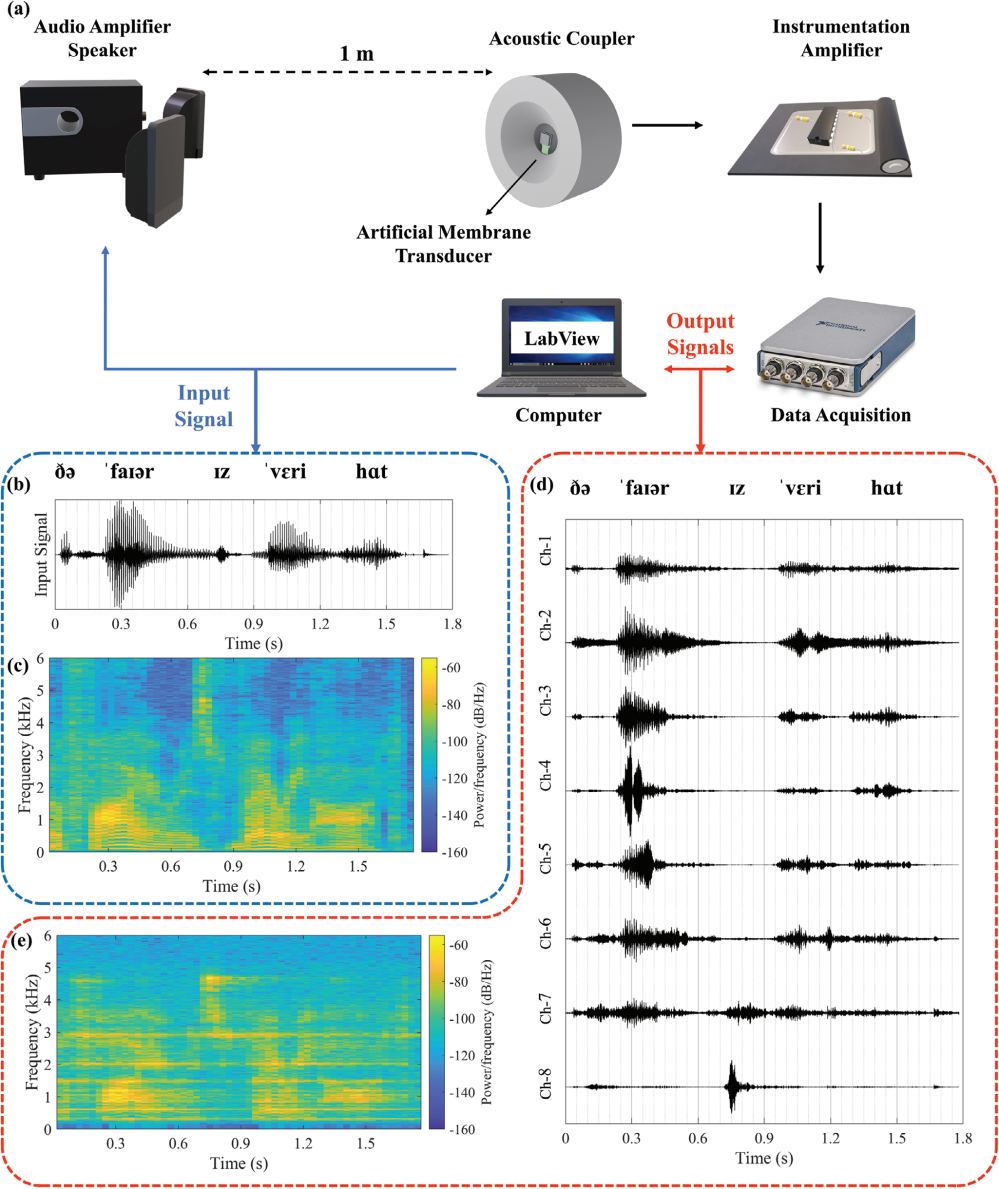
Speech test of the transducer. a Schematic of the test setup. b Time domain input waveform. c Spectrogram of the input signal. d Time domain output waveforms of each channel. e Spectrogram of the regenerated output signal.

**Figure** **4**a shows the test setup of speech tests. Recorded sentences were prepared by computer, amplified by an audio amplifier (PA210), and played by speaker (JBL Control One). Transducer was placed one meter away from the speaker, and sound level was measured with a sound level meter (IEC 651 Type II) next to the transducer. Output signal detection and recording have proceeded with the same system as acoustic characterization. Among the speech signals, transducer outputs in no signal condition were recorded to observe the noise performances of each channel. (Optical images of the test setups, characteristics of the acoustic holder designed to prevent any coincidence of incoming sound and standing waves, and details of the setups are shared in Supporting Information.)

## 3 Results

Output characteristics and performance of the transducer were obtained in realistic test environments. Figure 3a shows the acoustic test setup. In this setup, each parameter is controlled to get a practical environment for the application. Transducer was placed on an artificial membrane, and membrane vibration characteristic was measured with LDV. Figure 3d shows the vibration amplitudes of the membrane and measured umbo vibrations in the literature,^[^ ^35^, ^37^, ^38^, ^39^, ^40^ ^]^ and it shows that they are within the reported limits of the human tympanic membrane vibrations. The membrane was excited with insert earphones while sound level was controlled continuously, and the transducer was tested in the daily hearing range. Figure 3b shows the multi‐channel output performance in the hearing band under different excitation levels. In order to show the coverage of the channel, Figure 3c reveals the transducer performance under 70 dB SPL excitation. (Acoustic characteristics of the 8 channels are separately included in Supplementary Information to show the overall frequency selectivity of the transducer.)

The LDV results illustrate the average vibration amplitude of the transducer coupled with an artificial tympanic membrane to show the test system, and the obtained results fall within an acceptable range. However, it is crucial to acknowledge that the vibration modes of the eardrum could impact channel performance, particularly concerning the transducer's placement, and orientation. The vibration data presented in Figure 3d represents the average of vibration amplitudes measured at eight points on the transducer frame, with corresponding positions detailed in Figure S16 (Supporting Information). Table S1 (Supporting Information) tabulates measured values, and Figure S17 (Supporting Information) provides displacement values and mode shapes.

In order to qualify the performance of the transducer under daily conditions, we tested the transducer with a speech signal, “the fire is very hot / ðə ˈfaɪər ɪz ˈvɛri hɑt”. The average sound level of the signal was arranged ≈70 dB SPL to replicate the daily needs. The speech signal was selected from regular test sentences and recorded in noise‐free environment. Figure 4b,c show time domain waveform and spectrogram of the test signal. Sensed signal waveforms for each channel and spectrogram of recreated transducer output are shown in Figure 4d,e These test setups and results were also used for objective quality measurements.

Processed speech signal was tested with objective quality measures, which use mathematical expressions to qualify the outputs. Objective speech quality measures are classified according to the domain in which they operate, like time, spectral, and perceptual domains. Time domain measurements such as SNR and SNRseg (Segmental Signal‐to‐Noise Ratio) aim to reproduce the waveform and can usually be applied in the analog waveform. In the spectral domain, the comparison is more robust against a temporal misalignment and phase shifts between the original and encoded signals. Spectral‐domain measures are similar to speech codec design and depend on speech production patterns. A spectral comparison metric is developed by including logarithmic scaling, such as Log‐Likelihood Ratio, and Itakura‐Saito Distance features of the human speech production process, such as cepstral distance. Perceptual domain measurements such as PESQ and POLQ are based on the physiology of human auditory perception. They are the best for estimating the subjective quality of speech signals and transforming the signal into a perceptually modeled domain containing the human auditory. PESQ, the best‐known objective perceptual measurement method among all measures for speech quality measurement, was accepted as recommendation P.862 by ITU‐T for the measurement of speech quality.^[^ ^41^ ^]^ In the PESQ calculation, the original and degraded signals are equated to a standard listening level and filtered. The signals are time‐aligned to correct for time delays and then processed via auditory transform to obtain the loudness spectrum. The difference between loudness spectra, called disturbance, is calculated and averaged over time and frequency to produce an estimate of an overall quality rating score obtained on a five‐point Absolute Category Rating (ACR) scale.^[^ ^42^ ^]^ For the first time in the literature, outputs of an implantable transducer were characterized with the objective qualification method, PESQ. Transducer`s recreated output signals scored 3.42/4.5 in PESQ qualification method, and it represents “fair” in ACR scale. Recordings are available in the Supplementary Materials.

SNR measures the ratio of the strength of the signal of interest to the background noise level. In the case of an acoustic transducer, the signal is the sound produced by the source of interest, while the noise is the unwanted sounds present in the environment. To calculate SNR, transducer performance without input sound waves corresponding to noise recordings is needed. For this purpose, recordings were made for each channel with a duration of 3 s and a sampling rate of 25 600. The recordings were performed three times for each channel under no‐signal conditions. The power spectra analysis was carried out using the FieldTrip^[^ ^43^ ^]^ MATLAB toolbox. The recordings were divided into six windows of equal length and processed to suppress line noise and harmonics. An Infinite Impulse Response (IIR) two‐sided Discrete Fourier Transform (DFT) filter was applied to the recordings for this purpose. The power spectra were then calculated for each corresponding channel bandwidth using the “mtmconvol” method with a Hanning window. The Hanning window is a type of weighted function that is applied to the data before the Fourier transformation. This function is designed to minimize spectral leakage caused by the discontinuity at the edges of the data window.^[^ ^44^ ^]^ This reduces artifacts caused by discrete windows. A smoothing box with a frequency of 1 Hz was used to obtain a smooth power spectrum estimate. For each frequency, power spectra are averaged using three trials and eighteen windows. Finally, to calculate the SNR, the transducer output at 1 Pa was utilized. The time‐domain sound waveform was reconstructed using a sampling rate of 25 600, and the same power spectrum applications were applied as in the noise calculations. As a result, SNR levels of the channels varied between 66.8 and 84.2 dB. Table 1 shows the design parameters and output performances of each channel.

## 4 Discussion and Conclusion

An implantable transducer was developed by considering all the limitations and requirements of a CI, and its performance was validated. In the presented implantable system, ambient sound is sensed by the cantilevers via the piezoelectric effect. Studies in the literature test the transducer with single‐tone constant acceleration or sound test without any measurements and comparison of the vibration level with the hearing chain. Results presented in this study cover all relevant frequency ranges on an artificial tympanic membrane within the measured vibration levels on the human cadaver. Standard hearing test sentences are also used to excite the transducer. Then, outputs are evaluated with the objective qualification method, PESQ, and defined as the first merit and a milestone for middle‐ear implantable and acoustic transducers in the literature. For the first time in the literature, a middle ear implantable transducer was tested with objective qualification methods to the best of our knowledge. The obtained score of 3.42/4.5 was impressive for a multi‐channel filtering system under 70 dB speech signal excitation. Due to the nature of the mechanical filtering system, the quality factors of the channels are high, which causes output voltage differences depending on the frequencies. Therefore, regenerated speech signals have a slight distortion and ringing near the resonance frequency. This circumstance can be seen in the differences between spectrograms of the input (Figure 4c) and output (Figure 4e) signals. There are the main performance differences in the objective qualification method. However, during the stimulation of the auditory nerves, channels don`t work simultaneously. Measured signals at the resonance frequencies are observed due to the natural characteristic of the high‐Q filtering system. A small input can excite the channels. During the tests, output signals are recorded continuously. Contrarily, stimulation interfaces have stimulation strategies and use outputs of the channels in time intervals. They have specific orders, and waiting times provide a relaxation time for channels and prevent the ringing from reaching the user. For example, the most common strategy, CIS‐Continuous Interleaved Sampling, only processes one channel at a time. Therefore, the observed ringing effect will not be continuously included while stimulating the nerves, and the processed signal characteristic will be more compatible with the conventional systems. Differences in the amplitudes of the channels are not an issue for the application; the main point is the detection of the sound. Stimulation systems include a patient‐fitting feature to arrange each channel`s stimulation amplitude with respect to the user's feedback and condition of nerves.

Test environment is another factor; tests were not conducted in a noise‐free environment, and line noise in the system reduced the actual performance, especially in the low‐frequency channels. These imperfections caused the difference in the SNR test results at low‐frequencies and deviation from the linearities in high‐frequencies.

Points on the artificial tympanic membrane are exposed to various vibration levels at different frequencies, which shows that different modes are observed. Notably, the first three modes of the human tympanic membrane are significant at 400, 808, and 1849 Hz,^[^ ^45^ ^]^ and the addition of mass alters their prominent frequencies. In this experimental setup, the transducer is glued to the artificial tympanic membrane, causing slight variations in output results due to the placement, and orientation of the transducer. However, in real applications, the intention is to connect the transducer along a hearing chain from a single point using a connection apparatus. It is important to emphasize that this study strives to emulate a realistic testing environment by employing an artificial tympanic membrane. Mimicking the operational conditions and vibration levels of the ossicles, to the best of our knowledge, has not been achieved in existing literature. The average vibration level of the artificial tympanic membrane falls within the range of values measured in previous studies, indicating that the test method is the most convenient approach after cadaver experiments.

All references/grounds of the channels are connected to the common ground, and there is no crosstalk over the ground line. However, channels with high moment of inertia caused a slight effect on adjacent channels' output when they were excited with the resonance frequency at high sound pressure levels. This effect is also a result of gluing the transducer to the membrane. Using the connection apparatus to the hearing chain will eliminate this crosstalk.

Considering the surgical operation methods and optimizing the design with FEM simulations, the bilayer sound detector presented in this study has been designed and fabricated. Performances and dimensions of reported transducers in the literature are shown in **Table** **2**. This new generation transducer outperforms the previous detectors in terms of both sizes and output levels. The total volume of the transducer, excluding the packaging parts, was reduced by a factor of 60% while improving the output levels by 15 dB with an impressive objective qualification score and up to 84.2 dB signal‐to‐noise ratio. The distribution of channels and the layered architecture do not exhaust the considerations within this conceptual framework. In the system design, meticulous adjustments were made to individual channel parameters corresponding to distinct resonance frequencies. Contrary to previous investigations of our studies wherein uniform channel widths were maintained, the ensuing divergence in channel dimensions has engendered marked differentials in structural robustness, acoustic noise profiles, output metrics, and filter characteristics relative to the findings expounded in the present study.

**Table 2:** Acoustic test results of the transducer in the literature for similar applications.

Study	Channel Number	Frequency Range	Excitation	Output Level [mV]	Dimensions [mm^3^]
This Study	8	250–6000 Hz	100 dB	320.3	2 x (3 × 3 × 0.36)
[18]	8	250–5500 Hz	100 dB	50.7	5 × 5 × 0.62
[15]	8	2.9–12.6 kHz	101.7 dB	4.06	2.5 × 2.5 × 0.6
[14]	10	10v37 kHz	112.4 dB	3.55	≈6.5 × 6.5 × 0.35
[11]	16	300–700 Hz	1 g (>100 dB)	9.6	32 × 32 × 0.55
[17]	7	830–3600 Hz	120.1 dB	430	3–10 × 20 × 0.01
[20]	1	1325 Hz	100 dB	40	5 × 5 × 0.5

The expected success of implantable sound detectors would tremendously impact the patients' convenience, stigma, and lifestyle. The presented results prove that we are not far from the actual target. These performances can be improved more by studying vacuum packaging and forming application‐specific piezoelectric material. The presented study will be continued in two different aspects. First, the feasibility of the transducer‐interface electronics system will be tested with in vitro and in vivo methods. Results will show the dynamic range, power consumption, and sensitivity of the stimulation system of FICI while exciting RC load or cochlea. These data will underpin the feasibility of FICI system with middle ear implantable multi‐channel acoustic transducer and ultra‐low power interface electronics. In the second path, the packaging of the transducer will be examined. Vacuum packaging of the transducer is expected to improve the dynamic and frequency ranges of the transducer. The literature also lacks comprehensive studies on the placement and surgical techniques of a middle ear implantable system. Design parameters and restrictions are inferred from conventional systems and limited literature information. To optimize the system for FICI applications, a collaborative study with surgeons is necessary. Human cadaver studies need to be conducted to determine suitable locations, dimensions, and effects of additional mass on vibration characteristics, considering factors such as the connection aperture (as illustrated in Figure S7, Supporting Information), surgery tools, surgeon maneuverability, range of motion, and potential anatomical variations among individuals. Furthermore, application areas of the presented transducer are not limited to fully implantable cochlear implants; it can be used for low‐power sound detection systems: IoT, biometric detection, smart sensors, and security systems.

## Conflict of Interest

The authors declare no conflict of interest.

## Author Contributions

M.B.Y. was the main contributor to this work and responsible for transducer design, development, characterization, and data analysis. M.B.Y. and A.C.A. fabricated the transducers together. A.C.A. codesigned the study and reviewed the manuscript. H.K. contributed to the development of transducer concepts and critical revisions of the article. All authors have read and approved the final manuscript.

## Supporting information

Supporting Information

Supporting Information

## Data Availability

The authors declare that the data supporting the findings of this study are available within the paper. Raw data generated for this study are available from the corresponding author upon reasonable request.

## References

[1] F.‐G. Zeng , JASA Express Lett. 2022, 2, 077201. 36154048 10.1121/10.0012825

[2] Deafness and hearing loss, https://www.who.int/news‐room/fact‐sheets/detail/deafness‐and‐hearing‐loss (accessed: February 2023).

[3] M. K. Cosetti , S. B. Waltzman , Expert Rev. Med. Devices 2011, 8, 389. 21542710 10.1586/erd.11.12

[4] F. G. Zeng , S. Rebscher , W. Harrison , X. Sun , H. Feng , IEEE Rev. Biomed. Eng. 2008, 1, 115. 19946565 10.1109/RBME.2008.2008250 PMC2782849

[5] U.S. National Library of Medicine , Feasibility of the Mi2000 Totally Implantable Cochlear Implant in Severely to Profoundly Deaf Adults https://clinicaltrials.gov/ct2/show/NCT04571333 (accessed: April 2022).

[6] T. Narváez , Envoy Medical Early Feasibility Study of Breakthrough Fully Implanted Cochlear Implant Now Underway First Patient Receives Fully Implanted Acclaim® Cochlear Implant, www.envoymedical.com (accessed April 2023).

[7] R. J. S. Briggs , H. C. Eder , P. M. Seligman , R. S. C. Cowan , K. L. Plant , J. Dalton , D. K. Money , J. F. Patrick , Otol. Neurotol. 2008, 29, 114. 17898671 10.1097/MAO.0b013e31814b242f

[8] D. J. Young , M. A. Zurcher , M. Semaan , C. A. Megerian , W. H. Ko , IEEE Trans. Biomed. Eng. 2012, 59, 3283. 22542650 10.1109/TBME.2012.2195782

[9] J. Jang , J. H. Jang , H. Choi , Adv. Healthcare Mater. 2017, 6, 1700674.

[10] A. E. Hake , C. Zhao , W. K. Sung , K. Grosh , IEEE Sens. J. 2021, 21, 17703. 35177956 10.1109/jsen.2021.3085825 PMC8846575

[11] P. Udvardi , J. Radó , A. Straszner , J. Ferencz , Z. Hajnal , S. Soleimani , M. Schneider , U. Schmid , P. Révész , J. Volk , Micromachines 2017, 8, 311. 30400501 10.3390/mi8100311 PMC6190361

[12] N. Gao , Y. Z. Chen , F. L. Chi , T. Y. Zhang , H. D. Xu , H. Y. Kang , T. Z. Pan , Laryngoscope 2013, 123, 1506. 23625487 10.1002/lary.23618

[13] X. H. Jia , N. Gao , X. D. Xu , Y. Z. Wu , H. Y. Kang , F. L. Chi , Acta Otolaryngol. 2016, 136, 1248. 27388506 10.1080/00016489.2016.1201590

[14] J. Jang , S. Kim , D. J. Sly , S. J. O'Leary , H. Choi , Sens. Actuators, A 2013, 203, 6.

[15] J. Jang , J. Lee , S. Woo , D. J. Sly , L. J. Campbell , J. H. Cho , S. J. O'Leary , M. H. Park , S. Han , J. W. Choi , J. Hun Jang , H. Choi , Sci. Rep. 2015, 5, 1. 10.1038/srep12447 PMC4521187 26227924

[16] C. Zhao , K. E. Knisely , K. Grosh , in *19th International Conference on Solid‐State Sensors, Actuators and Microsystems* (TRANSDUCERS), IEEE, Kaohsiung, Taiwan, 2017, pp. 16–19.

[17] H. S. Wang , S. K. Hong , J. H. Han , Y. H. Jung , H. K. Jeong , T. H. Im , C. K. Jeong , B. Y. Lee , G. Kim , C. D. Yoo , K. J. Lee , Sci. Adv. 2021, 7, 5683. 10.1126/sciadv.abe5683 PMC7880591 33579699

[18] M. B. Yuksel , A. Koyuncuoglu , H. Kulah , IEEE Sens. J. 2022, 22, 3052.

[19] M. B. Yuksel , B. Ilik , A. Koyuncuoglu , H. Kulah , in IEEE SENSORS , IEEE, Montreal, QC, Canada, 2019, pp. 1–4.

[20] B. İlik , A. Koyuncuoğlu , Ö. Şardan‐Sukas , H. Külah , Sens Actuators A Phys 2018, 280, 38.

[21] A. Koyuncuoğlu , B. İlik , S. Chamanian , H. Uluşan , P. Ashrafi , D. Işık , H. Külah , Proceedings 2017, 1, 584.

[22] L. Beker , O. Zorlu , N. Goksu , H. Kulah , in *Transducers & Eurosensors XXVII*: The 17th Int. Conf. on Solid‐State Sensors, Actuators and Microsystems (TRANSDUCERS & EUROSENSORS XXVII), IEEE, Barcelona, Spain, 2013, pp. 1663–1666.

[23] H. Ulusan , A. Muhtaroglu , H. Kulah , IEEE Access 2019, 7, 132140.

[24] H. Külah , A Fully‐Implantable MEMS‐Based Autonomous Cochlear Implant | FLAMENCO Project | Fact Sheet | H2020 | CORDIS | European Commission, https://cordis.europa.eu/project/id/682756 (accessed: July 2016).

[25] H. Kulah , H. Ulusah , S. Chamanian , A. Batu , M. B. Ugur , M. B. Yuksel , A. M. Yilmaz , H. A. Yigit , A. Koyuncuoglu , O. Topcu , A. K. Soydan , IEEE 35th International Conf. on Micro Electro Mechanical Systems Conf. (MEMS), IEEE, Tokyo, Japan 2022, *2022‐January*, 396.

[26] MEDEL , SONATA Surgical Guideline vol. AW7695_5.0,https://www.medel.com/ (accessed: February 2021).

[27] H. T. Samuel , A. Lepcha , A. Philip , M. John , A. M. Augustine , Indian J. Otolaryngol. 2022, 74, 714. 10.1007/s12070-021-02512-0 PMC9411450 36032887

[28] Y. Suzuki , H. Takeshima , J. Acoust. Soc. Am. 2004, 116, 918. 15376658 10.1121/1.1763601

[29] B. S. Wilson , Cochlear Implants: Auditory Prostheses and Electric Hearing, (Eds.: F. G. Zeng , A. N. Popper , R. R. Fay ), Springer Science, New York, 2004, 14.

[30] S. Nishihara , H. Aritomo , R. L. Goode , Otolaryngol Head Neck Surg. 1993, 109, 899. 8247572 10.1177/019459989310900520

[31] N. B. H. Croghan , S. I. Duran , Z. M. Smith , J. Acoust. Soc. Am. 2017, 142, EL537. 29289062 10.1121/1.5016044

[32] C. Garnham , M. O'Driscoll , R. Ramsden , S. Saeed , Ear Hear 2002, 23, 540. 12476091 10.1097/00003446-200212000-00005

[33] L. M. Friesen , R. V. Shannon , D. Baskent , X. Wang , J. Acoust. Soc. Am. 2001, 110, 1150. 11519582 10.1121/1.1381538

[34] K. Arora , R. Dowell , P. Dawson , Modern Speech Recognition Approaches with Case Studies, (Eds.: S. Ramakrishnan ), IntechOpen, London, England, 2012, Ch. 10.

[35] D. J. Young , M. A. Zurcher , T. Trang , C. A. Megerian , W. H. Ko , ENT‐Ear, Nose & Throat Journal 2010, 89, 21. 20155695

[36] A. Koyuncuoğlu , D. Işık Akçakaya , Ö. Ş. Sukas , H. Külah , Micro Nano Eng. 2022, 16, 100153.

[37] P. Ashrafi , D. Işık , H. Külah , J. Hear Sci. 2018, 2, 8.

[38] X. Zhang , X. Guan , D. Nakmali , V. Palan , M. Pineda , R. Z. Gan , J. Assoc. Res. Otolaryngol. 2014, 15, 867. 25106467 10.1007/s10162-014-0482-8 PMC4389959

[39] R. L. Goode , G. Ball , S. Nishihara , Am. J. Otolaryngol. 1993, 14, 247. 8372921

[40] R. L. Goode , G. Ball , S. Nishihara , K. Nakamura , Am. J. Otolaryngol. 1996, 17, 813. 8915406

[41] P.862 : Revised Annex A – Reference implementations and conformance testing for ITU‐T Recs P.862, P.862.1 and P.862.2. https://www.itu.int/rec/T‐REC‐P.862‐200511‐I!Amd2/en (accessed: March 2023).

[42] P. Lingapuram , Master Thesis, Blekinge Institude of Technology, 2012.

[43] R. Oostenveld , P. Fries , E. Maris , J. M. Schoffelen , Comput. Intell. Neurosci. 2011, 2011, 156869. 21253357 10.1155/2011/156869 PMC3021840

[44] O. M. Essenwanger , Elements of Statistical Analysis, Elsevier, Amsterdam‐London‐Newyork 1986.

[45] M. von Witzleben , T. Stoppe , T. Ahlfeld , A. Bernhardt , M. L. Polk , M. Bornitz , M. Neudert , M. Gelinsky , Adv. Healthcare Mater. 2021, 10, 2002089. 10.1002/adhm.202002089 PMC11468533 33506636

