# Supplementary material for: Piezoelectric Multi‐Channel Bilayer Transducer for Sensing and Filtering Ossicular Vibration
Source: Adv Sci (Weinh). 2024 Feb 21;11(16):2308277. doi: 10.1002/advs.202308277 (PMC11040378; doi:10.1002/advs.202308277)
Supplement: Supplementary file 1 — Supporting Information [file ADVS-11-2308277-s001.pdf]

ADVANCED SCIENCE
Open Access

Supporting Information

for *Adv. Sci.*, DOI 10.1002/advs.202308277

Piezoelectric Multi-Channel Bilayer Transducer for Sensing and Filtering Ossicular Vibration

*Muhammed Berat Yüksel, Ali Can Atik and Haluk Külah**

Supporting Information

Piezoelectric Multi-Channel Bilayer Transducer for Sensing and Filtering Ossicular Vibration

Muhammed Berat Yüksel, Ali Can Atik and Haluk Külah

1. Simulation and Design of the Transducer

Transducer was designed under the following restrictions:

- Volume of the middle ear: ~1 cm^3,
- Average area of the eardrum: ~9 x 10 mm^2,
- Surgery opening: ~4.7 mm,
- Maximum total mass: 25 mg,
- Frequency range: 250-4000 Hz human voice & above is useful for speech in noise,
- Channel number: 8-channel,
- Channel distribution: 300-600-900-1200-1600-2200-3200-4800 Hz,
- Output level: 100 µV for ultra-low power applications.

Beam structure was modeled with COMSOL Multiphysics. Design parameters of piezoelectric beam and how they are defined are listed as:

- PZT thickness: 1 µm - Suggested thickness for a similar application by studies of the supplier company,
- Beam thickness: 10 µm – Calculated for required frequencies and limited by maximum stress on the structure,
- Mass thickness: 350 µm - Calculated for required frequencies and limited by maximum stress on the structure,
- Length of beam: Simulation and limited by maximum length,
- Length of mass: Simulation and limited by maximum loading,
- Width of beam: Simulation and limited by noise and maximum width,
- Length of PZT: Simulation and limited by electrical properties and fabrication,

Assumptions during the simulations:

- Metal and isolation layers were omitted,
- Ideal load was connected ($R_L=\infty$),
- Air drag damping was excluded,
- Piezoelectric layer is assumed to cover the given area in other words, mask margins, 10 µm in all directions, were omitted,
- Electrical damping due to the input characteristics of the interface circuit was excluded.

The meshing of the simulation model has an impact on both accuracy and computational time. Smaller meshes increase the accuracy. However, this approach increases computation time, especially for thin-layered structures. Since metal and insulating layers' material characteristics and thicknesses barely affect the output characteristics, they are disregarded. Also, meshing thin layers with tetrahedral meshes is not efficient. This meshing strategy increases the mesh number a lot or reduces the accuracy due to the lack of stress level calculation on the material. Therefore, we arrange the mesh size and type of the model manually until it converges.

During simulations, the vibration characteristics of the channels were simulated under constant acceleration to achieve the required output signal for ultra-low-power neural interface electronics. The acoustic performance of the transducer was estimated by comparing the vibration levels of in vitro tests in the literature. Figure S1 shows the simulation strategy to design a multi-channel system. There are four main steps for this application.

1. Define beam/mass thickness ratio to choose SOI wafer,
2. Find beam/mass length ratio,
3. Find beam/piezo length ratio,
4. Choose lengths and widths of channels.

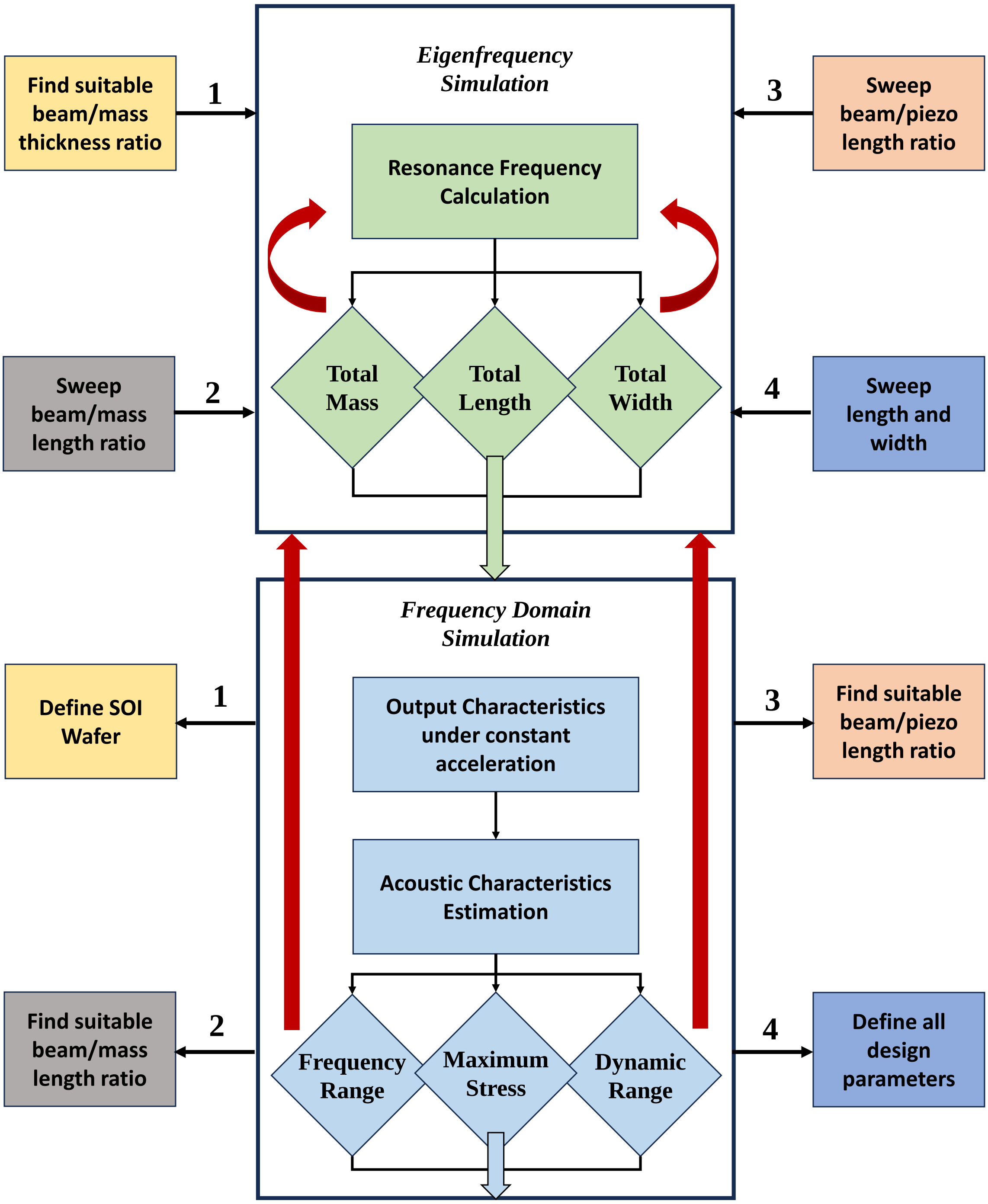

Figure S1. Simulation strategy flow of the transducer.

2. Test Setups

The transducer has been tested on the setup in Figure S2 for acoustic tests and in Fig. S3 for speech tests. The Transducer was placed on an artificial tympanic membrane designed for by group, and electrical connections were carried on a flexible substrate. During the acoustic tests, a pure tone signal was generated by a signal generator (Keysight 33522B), amplified by an audio amplifier (DENON PMA520), and the membrane was excited by an insert earphone (Etymotic Research, ER-2). On the other hand, recorded speech signals were processed in a computer, amplified by an audio amplifier (PA210), and given out by a speaker (JBL Control One) with a one-meter distance during the speech test. Excitation levels are arranged by the sound level meter (IEC 651 Type II) calibrated in a 2-cc acoustic coupler. The output voltage level of the transducer was amplified by a battery-powered instrumentation amplifier (INA121P), detected by Data Acquisition Board (DAQ – NI cDAQ-9174), and processed by LabView software.

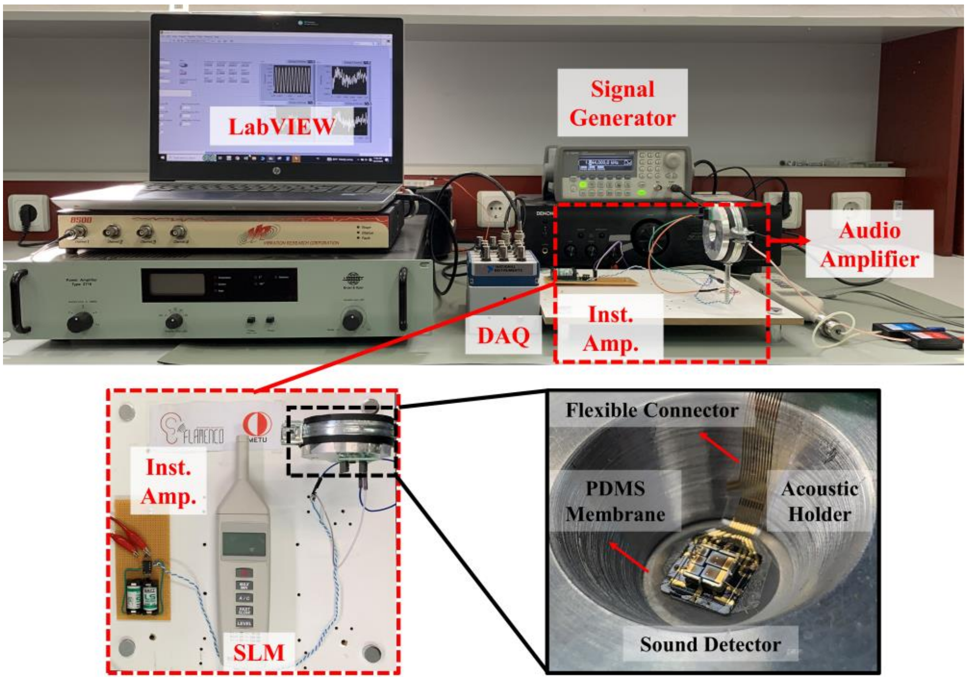

Figure S2. Acoustic test setup for the multi-channel transducer.

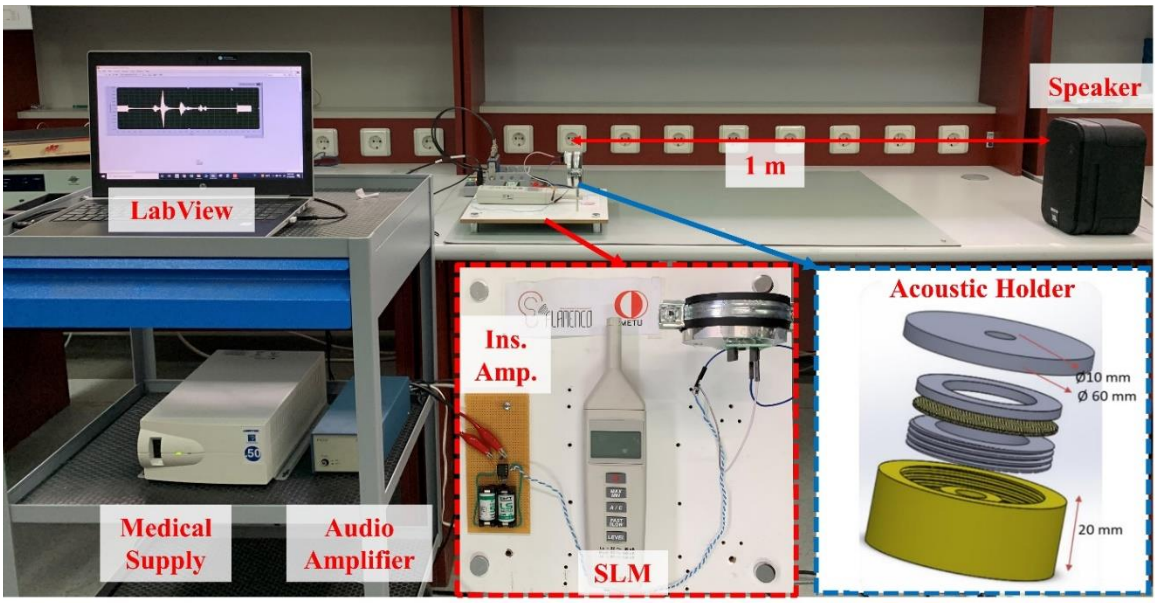

Figure S3. Test setup of the transducer for speech tests.

The vibration level of the membrane and transducer was measured on the test setup in Figure S4. Similar to the acoustic test, a pure tone signal was generated by a signal generator (Keysight 33522B), amplified by an audio amplifier (DENON PMA520), and the membrane was excited by an insert earphone (Etymotic Research, ER-2). Excitation levels are arranged by the sound level meter (IEC 651 Type II) calibrated in a 2-cc acoustic coupler. Vibration level of the transducer and membrane couple were measured from 8 different locations by using Laser Doppler Vibrometer (LDV, Polytec, NLV-2500), and the average value is accepted as vibration level.

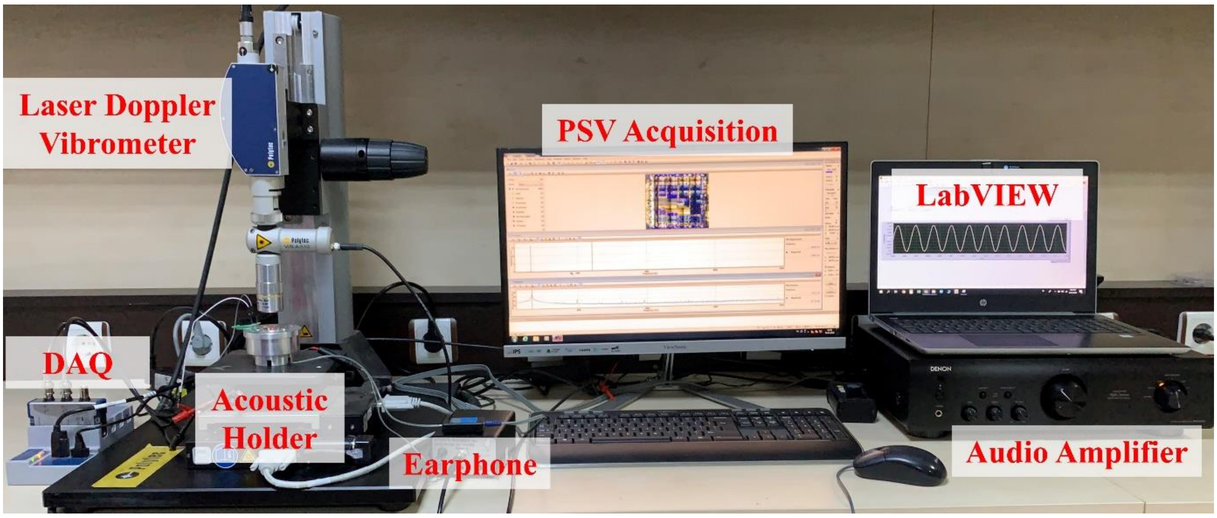

Figure S4. Test setup for the vibration level measurement of the membrane and transducer.

Previously, the acoustic holder was designed in our group to perform similar characteristics to the human ear canal. The ear canal can amplify the incoming acoustic wave pressure at specific frequencies. FEM simulations are used to simulate the artificial ear canal in order to mimic this characteristic and arrange the dimensions of the holder. Figure S5 shows the FEM simulation results for the dimensions of the acoustic holder.

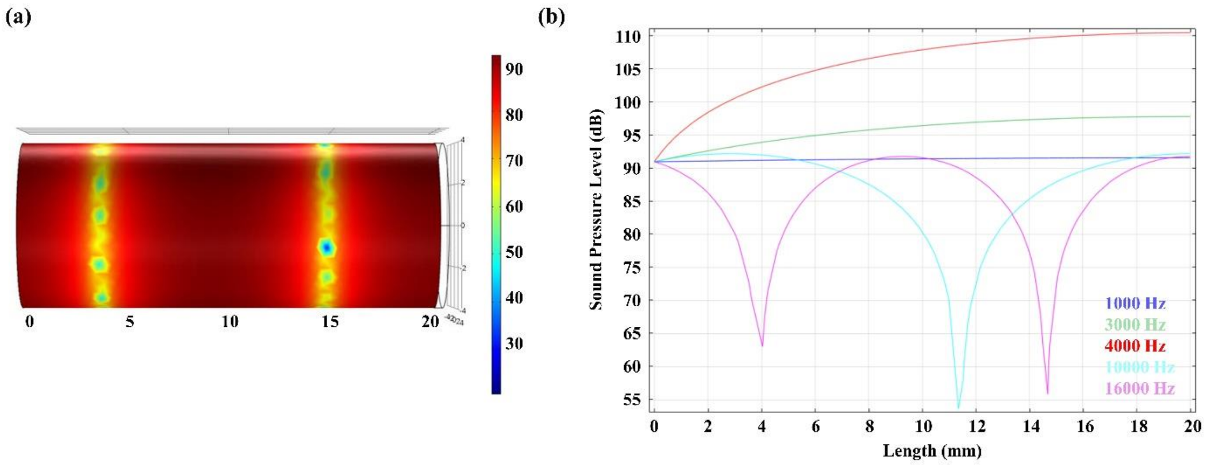

Figure S5. FEM characterization of the acoustic holder [37].

3. Optic Images of the Transducer

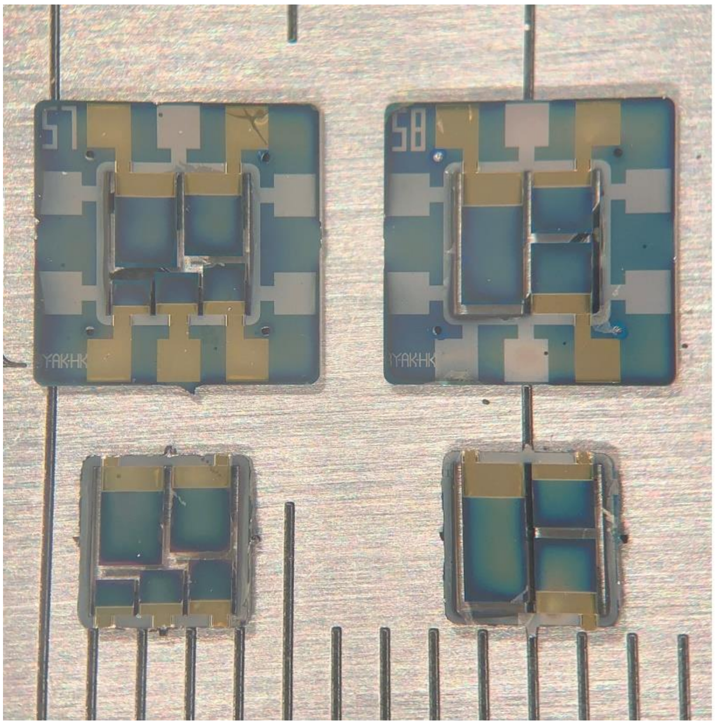

Figure S6. Optic image of the two layers of the transducer with and without test frames.

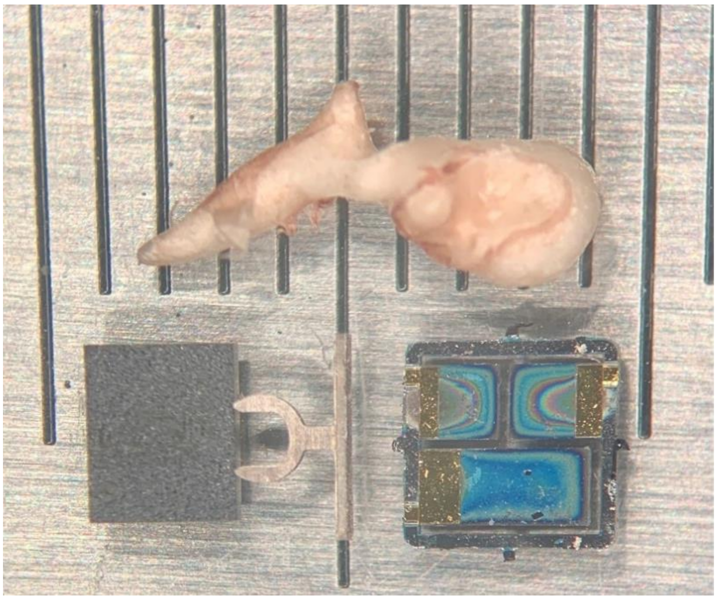

Figure S7. Optic image of human malleus, one layer of the transducer, accelerometer (STM-LSM6DSL) for size comparison and titanium clips for application.

4. Acoustic Characteristic of 8-Channel

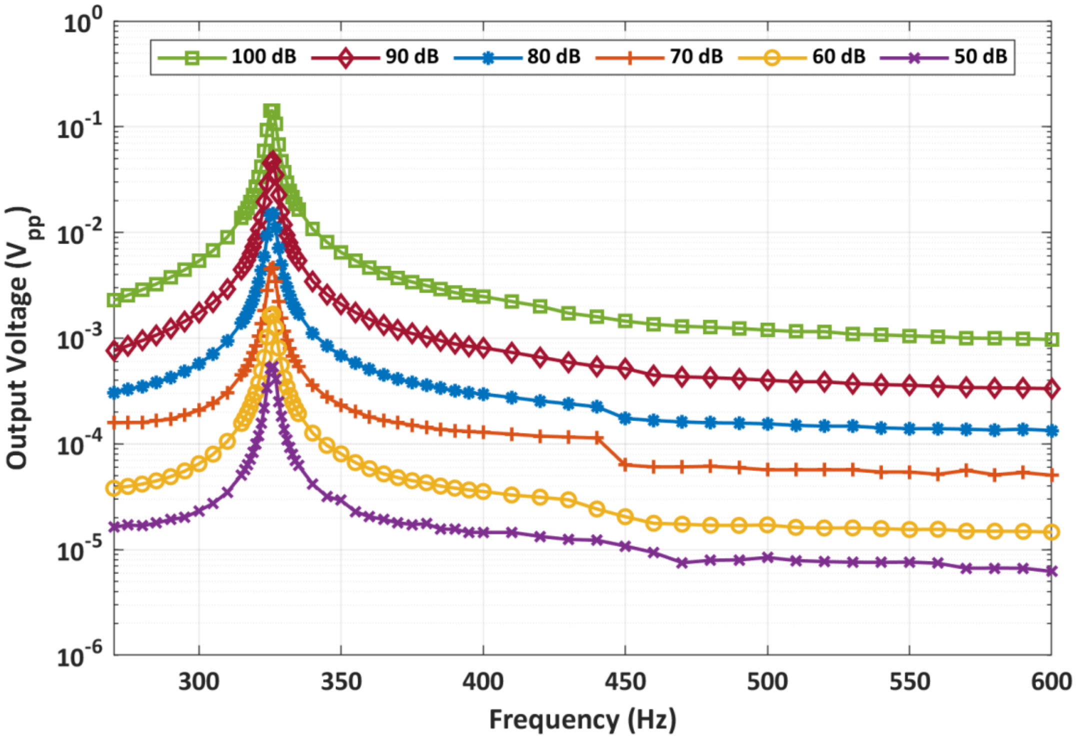

Figure S8. Acoustic Characteristic of the 1st channel.

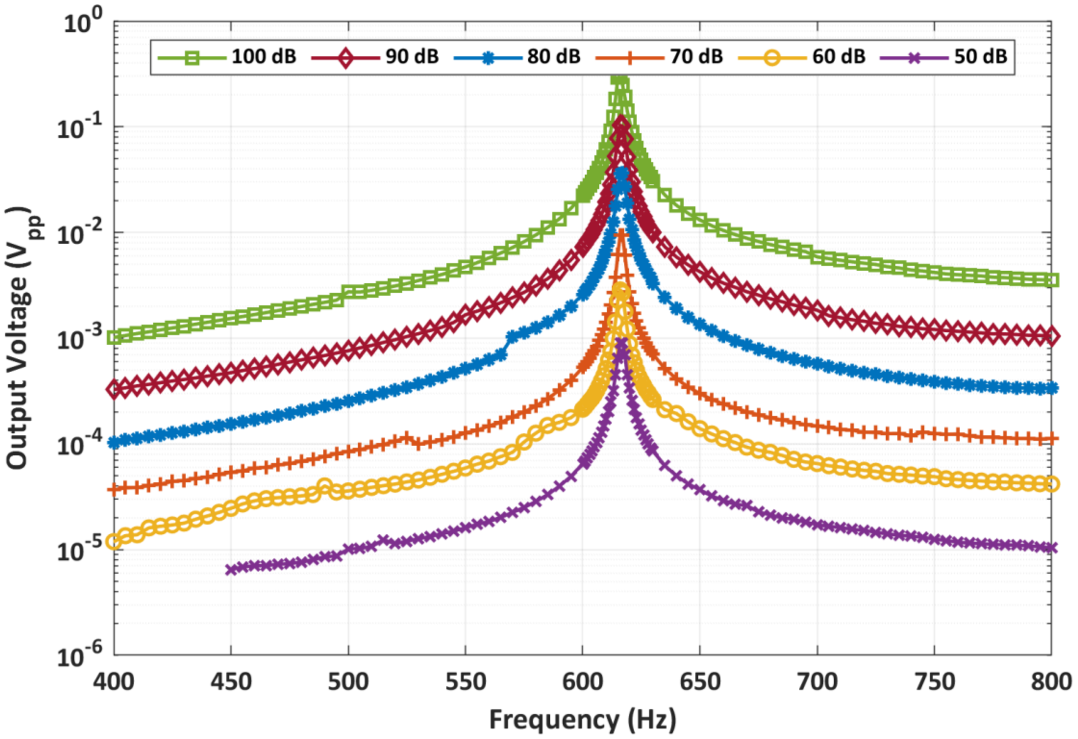

Figure S9. Acoustic Characteristic of the 2nd channel.

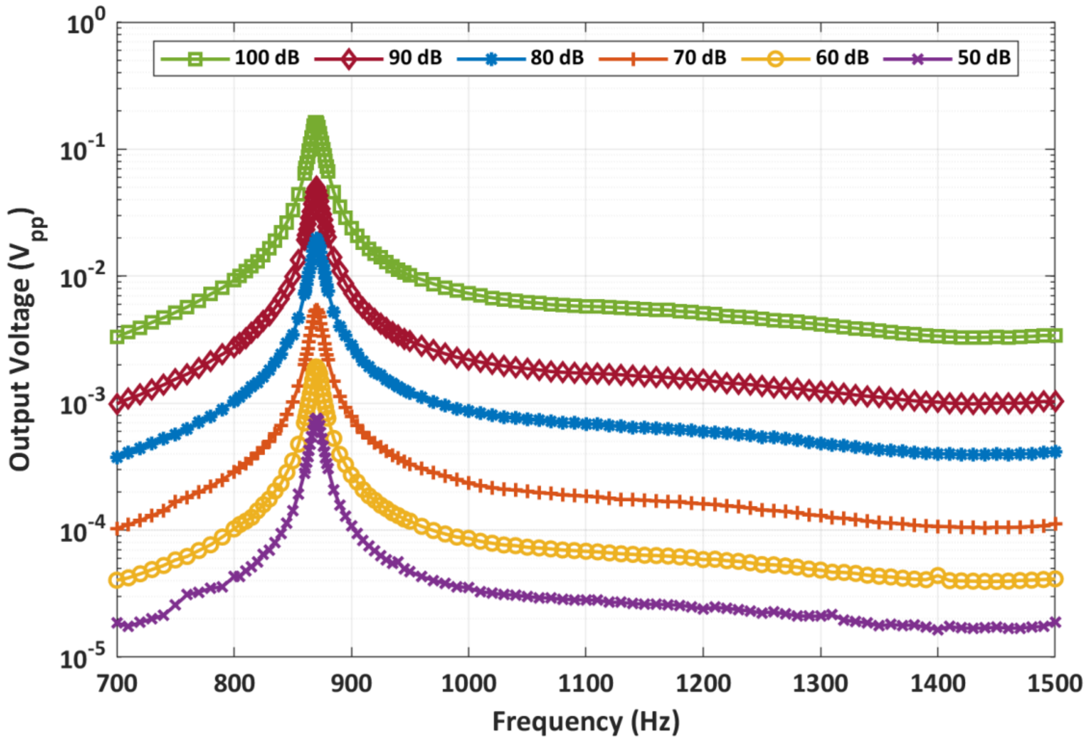

Figure S10. Acoustic Characteristic of the 3rd channel.

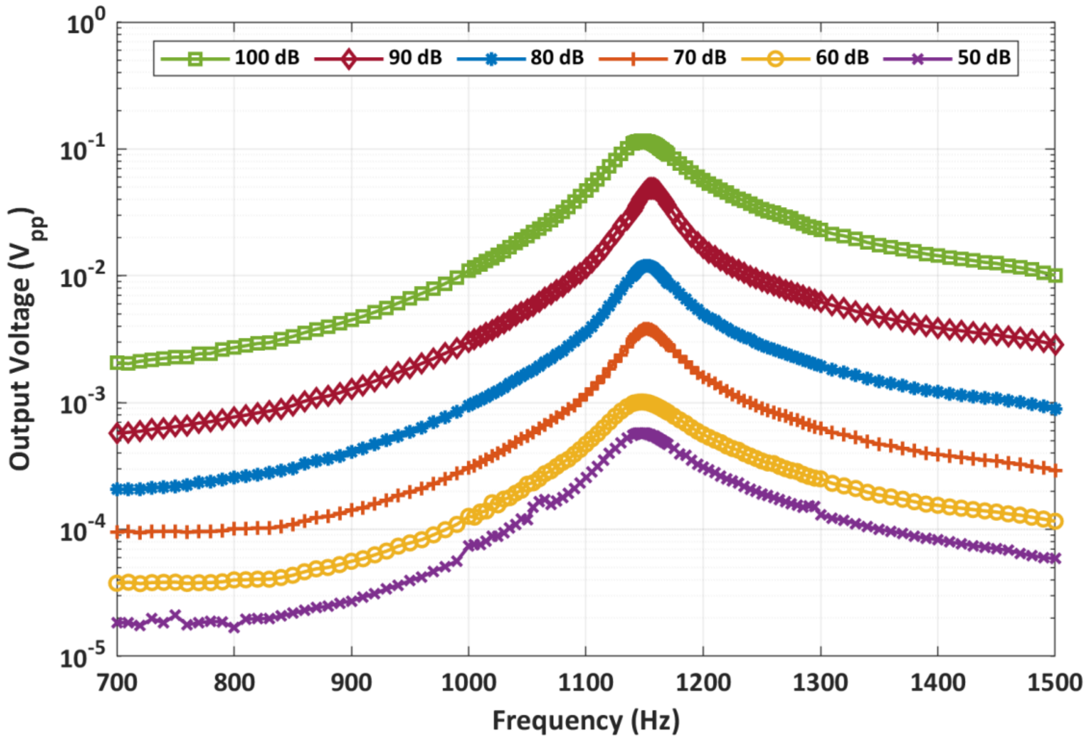

Figure S11. Acoustic Characteristic of the 4th channel.

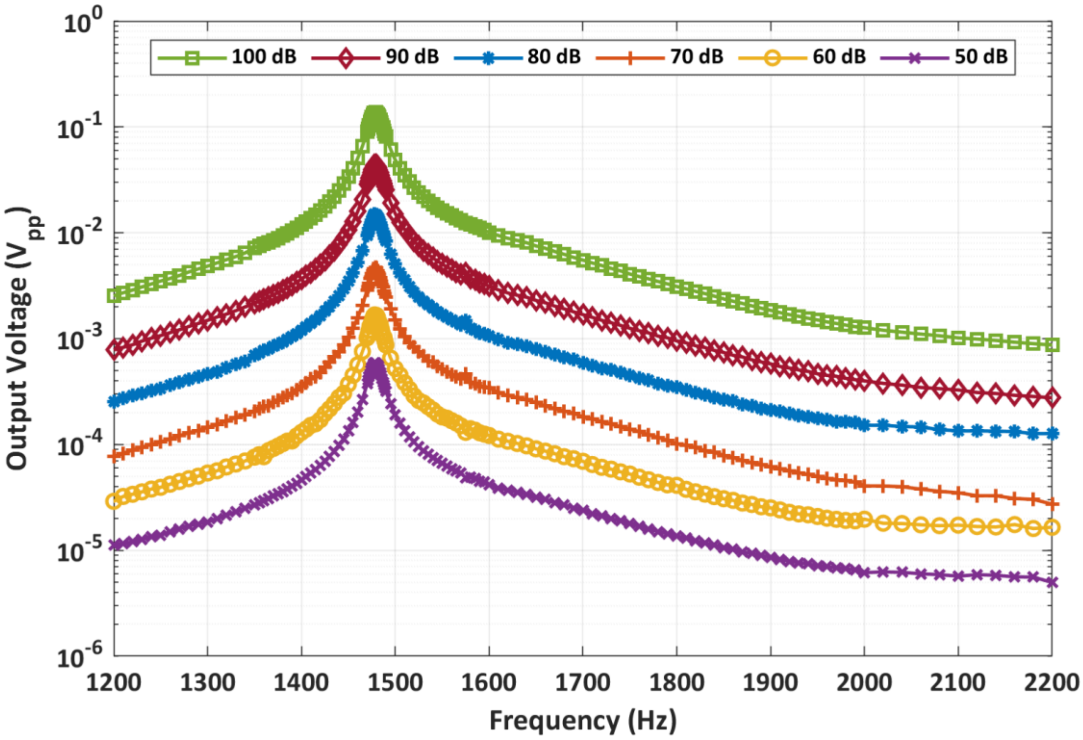

Figure S12. Acoustic Characteristic of the 5th channel.

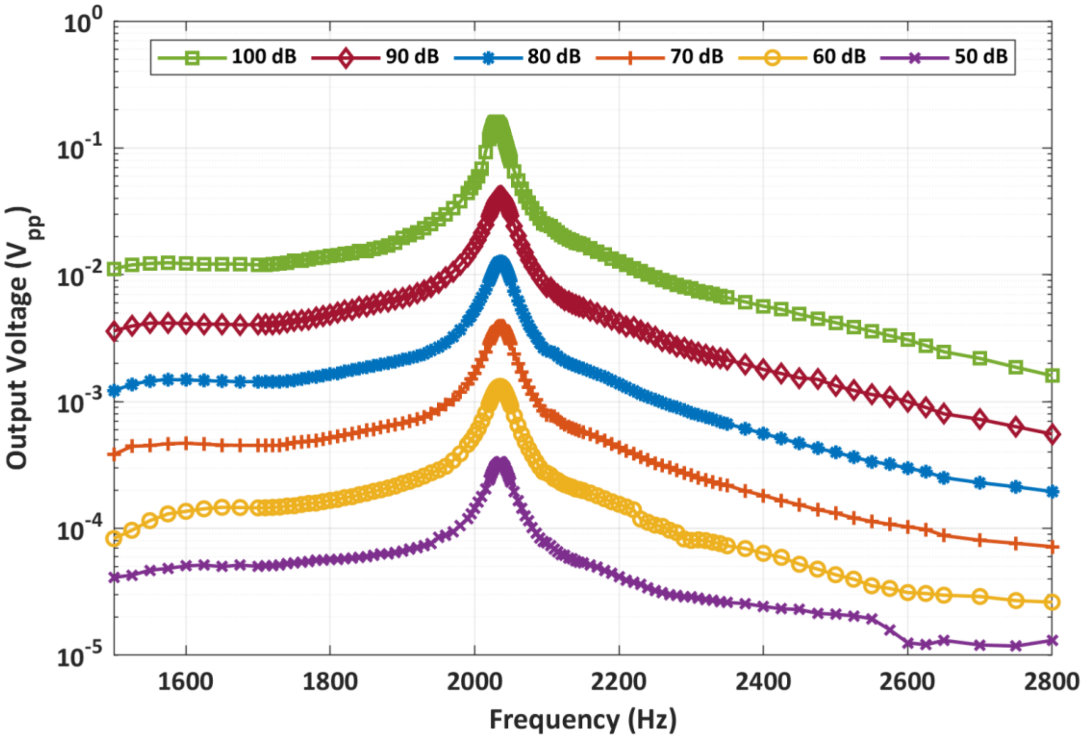

Figure S13. Acoustic Characteristic of the 6th channel.

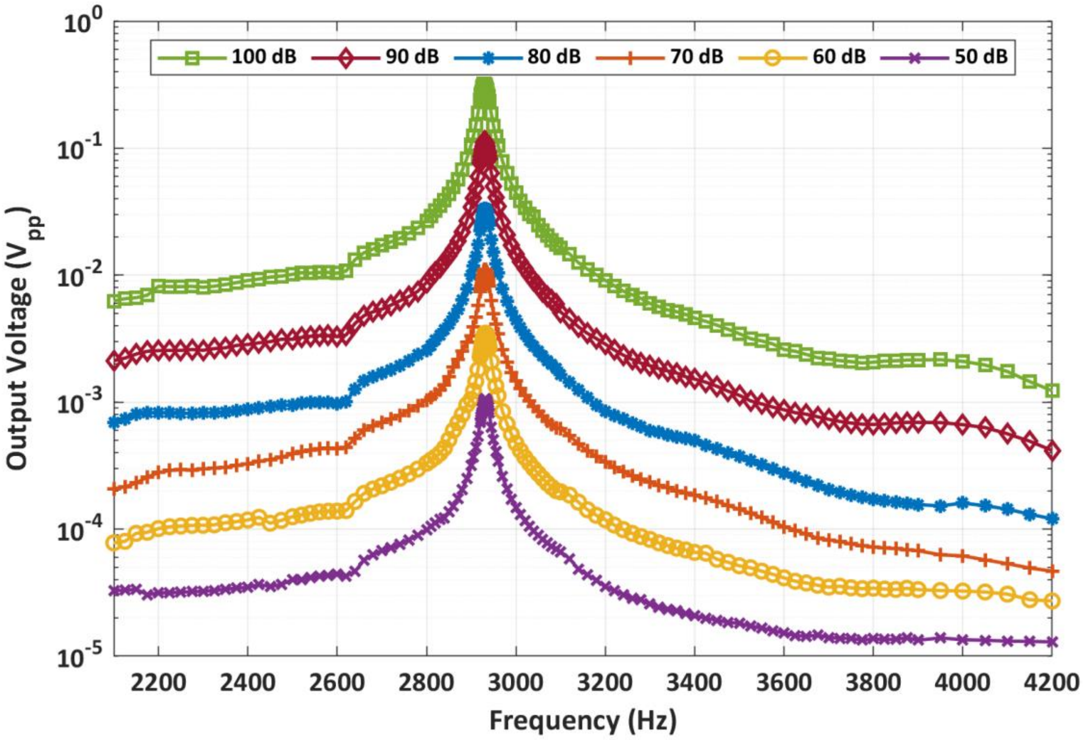

Figure S14. Acoustic Characteristic of the 7th channel.

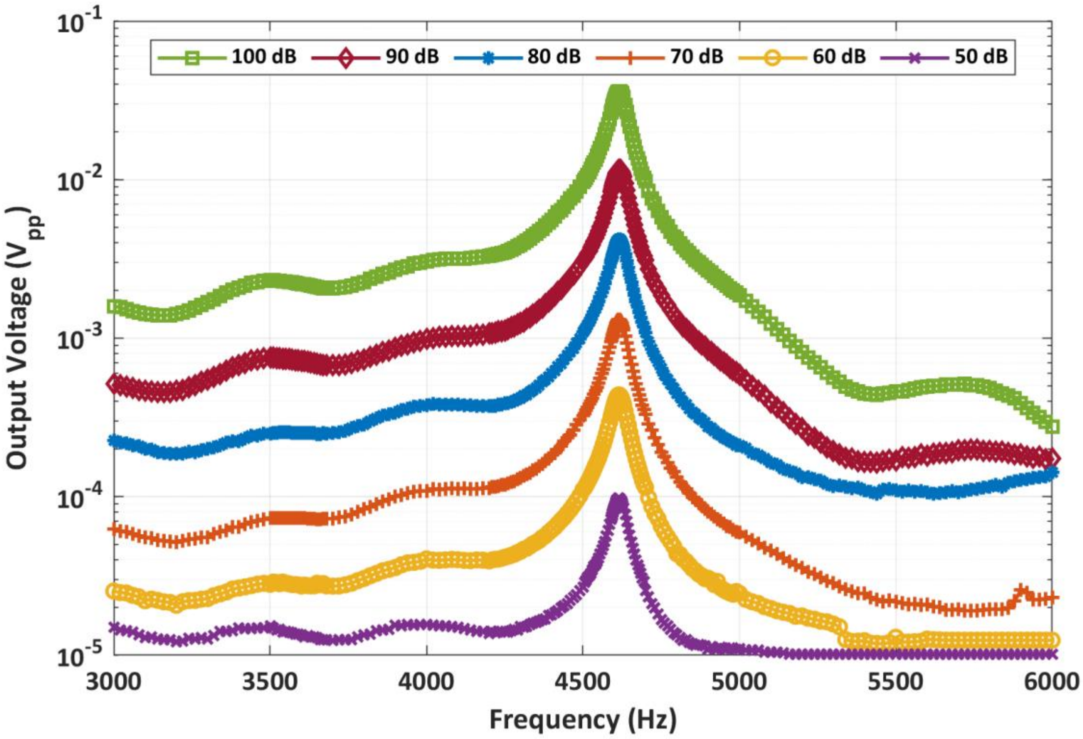

Figure S15. Acoustic Characteristic of the 8th channel.

5. Vibration and Acceleration Levels of the Artificial Tympanic Membrane and Transducer

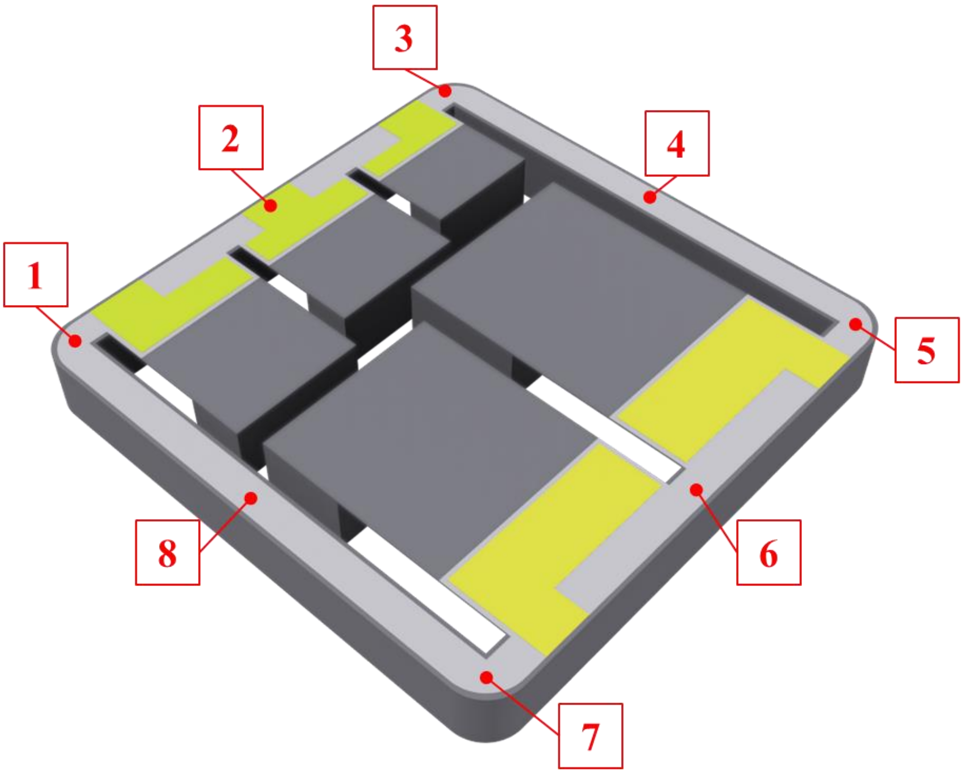

Figure S16. Vibration measurement points of the artificial tympanic membrane and transducer.

Table S1. Acceleration and displacement measurements of the artificial tympanic membrane and acoustic transducer under 80 dB excitation.

	Frequency	1	2	3	4	5	6	7	8	Average
Acceleration (m/s^2)	300	0.085	0.076	0.066	0.073	0.080	0.090	0.099	0.092	0.083
	600	0.724	0.690	0.649	0.690	0.729	0.768	0.805	0.727	0.722
	900	1.547	1.309	1.128	1.508	1.576	1.761	1.983	1.741	1.569
	1200	6.532	5.626	4.630	4.583	4.547	5.595	6.593	6.622	5.591
	1600	6.896	6.419	6.444	6.838	7.825	6.690	5.816	6.308	6.655
	2200	6.194	5.233	4.443	5.645	6.899	7.696	8.481	7.221	6.477
	3200	4.757	3.980	3.201	3.766	4.370	5.027	5.771	5.391	4.533
	4800	1.233	1.957	2.740	2.968	3.738	3.350	3.115	2.084	2.648
Displacement (nm)	300	23.894	21.358	18.551	20.425	22.380	25.250	27.941	25.964	23.220
	600	50.950	48.544	45.636	48.522	51.266	54.010	56.614	51.133	50.834
	900	48.365	40.936	35.269	47.171	49.286	55.061	61.998	54.442	49.066
	1200	114.906	98.970	81.440	80.620	79.981	98.426	115.969	116.486	98.350
	1600	68.237	63.518	63.761	67.655	77.426	66.194	57.550	62.415	65.845
	2200	32.415	27.389	23.253	29.544	36.108	40.279	44.385	37.792	33.896
	3200	11.767	9.845	7.918	9.317	10.811	12.436	14.275	13.334	11.213
	4800	1.355	2.152	3.013	3.263	4.110	3.683	3.425	2.291	2.911

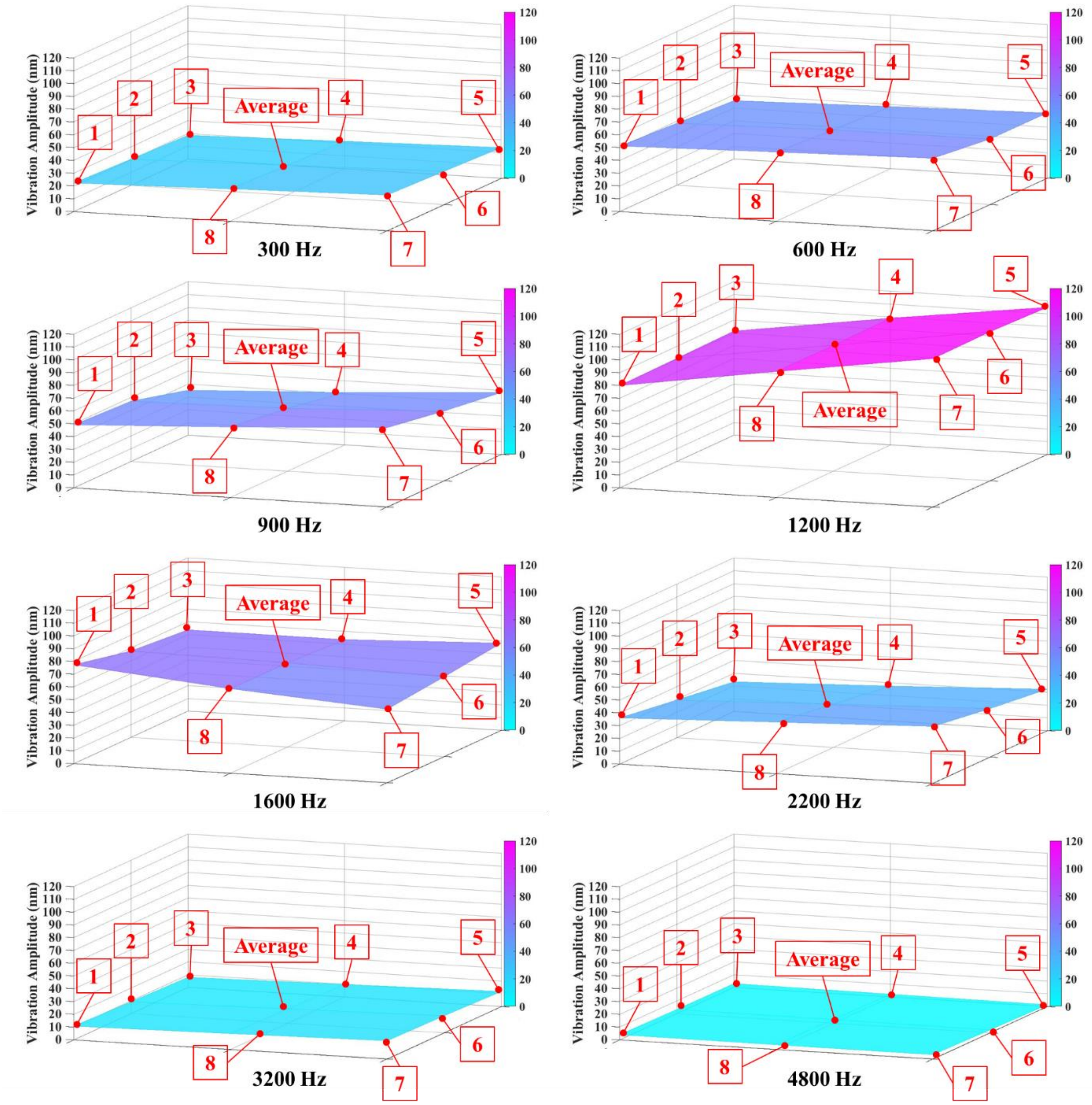

Figure S17. Displacement and mode shapes of the artificial tympanic membrane and transducer couple measured at 8 points of the frame and average value under 80 dB SPL at aimed frequencies.
